# Forensic LC–MS/MS Monitoring of Anticoagulant Rodenticide Poisoning in Cats Over a Half‐Decade

**DOI:** 10.1002/vms3.71209

**Published:** 2026-09-10

**Authors:** HyunYoung Chae, Jae‐Won Byun, So‐Jeong Yim, Bok‐Kyung Ku, Tae‐Wan Kim, JeongWoo Kang

**Affiliations:** ^1^ Animal Disease Diagnosis Division Animal and Plant Quarantine Agency (APQA) Ministry of Agriculture Food and Rural Affairs Gimcheon‐si Republic of Korea; ^2^ Laboratory of Veterinary Physiology College of Veterinary Medicine Kyungpook National University Daegu Republic of Korea

**Keywords:** coumatetralyl, gastric contents, QTRAP, QuEChERS, toxicovigilance

## Abstract

**Background:**

Anticoagulant rodenticides (ARs) are important toxicants in veterinary forensic investigations involving suspected poisoning in cats.

**Objectives:**

This study aimed to develop and validate a standardized LC–MS/MS‐QTRAP method for the simultaneous identification and quantification of eight ARs in gastric matrices from cats and to assess its applicability to veterinary forensic toxicology.

**Methods:**

Automated sample preparation was implemented to reduce inter‐analyst variability. Dual confirmation using multiple reaction monitoring (MRM) and enhanced product ion (EPI) spectra was applied to improve identification reliability and minimize false‐positive results caused by structurally related compounds. The method was applied to 747 cat forensic toxicology cases submitted between January 2020 and August 2025, in which gastric tissue or gastric contents were available for analysis.

**Results:**

ARs were detected in 2.5% of cases (19/747). Coumatetralyl was the most frequently detected AR, followed by flocoumafen and bromadiolone. Flocoumafen was detected despite not being an officially registered active ingredient in Korea, highlighting the need for broad multi‐analyte screening.

**Conclusions:**

The standardized LC–MS/MS‐QTRAP method enabled reliable detection and quantification of ARs in gastric matrices from cats. This approach provides useful forensic evidence of recent AR exposure in suspected poisoning cases and may support improved toxicovigilance for registered and unregistered rodenticides.

## Graphical abstract

This study validates a optimized LC–MS/MS method for multi‐analyte toxicological screening in cat gastric samples, eliminating complex manual processing by using automated sample preparation and demonstrating reliable compound separation and detection through simplified extraction and chromatographic analysis.

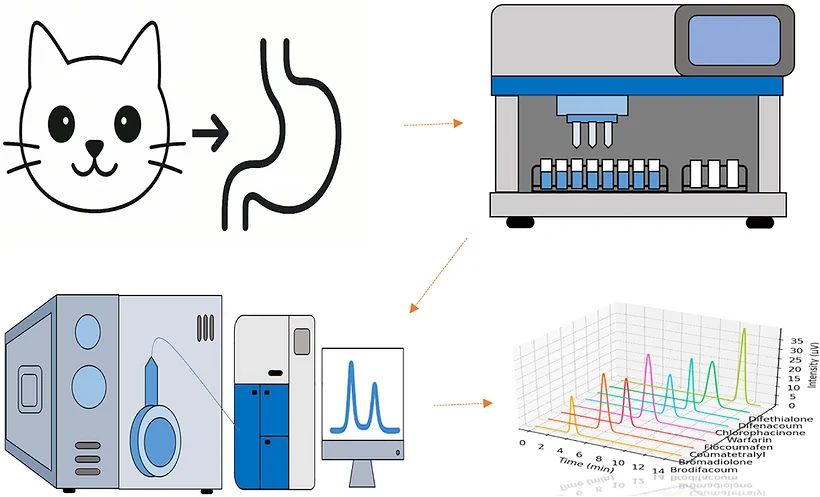

## 1 Introduction

Anticoagulant rodenticides (ARs) are widely used to control rodent populations in agricultural and urban environments. ARs inhibit the synthesis of vitamin K‐dependent coagulation factors (II, VII, IX and X), disrupting blood coagulation and causing internal haemorrhage, ultimately leading to death (Berny et al. 1995; Mount 1988). ARs are classified as first‐ or second‐generation compounds according to their potency, persistence and toxicokinetic properties. First‐generation anticoagulants (e.g., warfarin, chlorophacinone, coumatetralyl and diphacinone) have relatively low toxicity, short half‐lives and require repeated ingestion to reach lethal doses. In contrast, second‐generation anticoagulants (e.g., bromadiolone, brodifacoum, flocoumafen and difethialone) exhibit higher potency and longer half‐lives, potentially causing fatal poisoning after a single exposure. Because of their prolonged persistence in biological systems, second‐generation anticoagulants pose an increased risk of bioaccumulation and secondary poisoning in non‐target species, including companion animals. In addition, their high environmental persistence raises concerns regarding their long‐term accumulation, which can adversely affect companion animals and wildlife (Sánchez‐Barbudo et al. 2012; Vandenbroucke, Bousquet‐Mélou, et al. 2008).

Clinically, AR poisoning primarily manifests as haemorrhagic disorders, including pale mucous membranes, extensive subcutaneous haemorrhage, pulmonary or gastrointestinal bleeding and severe internal haemorrhage (Griggs et al. 2016). Because exposure is often unwitnessed and clinical signs may be delayed, diagnosis in cats can be challenging.

Although these rodenticides are effective at controlling rodent infestations, concerns regarding their safety have arisen because of their toxicity to non‐target organisms, particularly companion animals such as dogs and cats (Tauer 2025; American Society for the Prevention of Cruelty to Animals 2025). As the living environments of companion animals increasingly overlap with areas where rodenticides are used, cases of rodenticide poisoning in companion animals have been continuously reported in Korea.

According to studies conducted between 2019 and 2022 by the Animal and Plant Quarantine Agency and National Forensic Service both of which are government‐affiliated institutions in South Korea, ARs have been identified as some of the most frequently detected toxic substances in poisoning cases involving companion animals. These findings suggest that the widespread use of and environmental exposure to rodenticides significantly contribute to the increasing incidence of companion animal poisoning, underscoring the need for continuous monitoring and regulatory measures (Kang et al. 2023).

Furthermore, according to reports from the Animal Poison Control Center in the United States (ASPCA Animal Poison Control Center 2024, 2025), rodenticides are among the most frequently encountered toxic agents in companion animals. As rodenticide use continues to increase, concerns among companion animal owners have increased, highlighting the need for systematic research and preventive measures (Heestermans et al. 2022; Lefebvre et al. 2017). AR poisoning in companion animals can occur through various routes of exposure. The ingestion of rodenticide baits, ingestion of improperly placed bait or ingestion of poisoned rodents are the primary causes. Animals exhibiting exploratory or scavenging behaviour, such as dogs and cats, are particularly vulnerable to such exposure. Furthermore, early signs of rodenticide exposure in companion animals may go unrecognized by their owners, making early diagnosis difficult. In cats, exposure is often unwitnessed because rodenticides placed in areas accessible to rodents may also be inadvertently accessible to them. In some cases, such exposure may even be deliberate rather than accidental (Bille et al. 2016; Kohn et al. 2003; Gallocchio et al. 2014). These circumstances highlight the need for analytical methods capable of directly identifying specific AR compounds in biological samples.

LC–MS/MS has been introduced as an analytical technique to achieve a more precise diagnosis of AR poisoning. LC–MS/MS enables the highly sensitive detection of trace amounts of rodenticide residues in biological matrices, such as gastric contents, liver tissue and blood. In contrast to traditional coagulation tests, LC–MS/MS allows the direct identification and quantification of toxic compounds (Vandenbroucke, Desmet, et al. 2008; Seljetun et al. 2018). In particular, it facilitates the detection of highly potent second‐generation anticoagulants, such as bromadiolone and brodifacoum, even at low concentrations, making it an essential tool for confirming AR poisoning (Zhu et al. 2013). Currently, forensic toxicology widely employs instrumental analysis methods, such as LC–MS/MS, for criminal investigations, cause‐of‐death determinations and environmental toxicology assessments, using well‐established systems for detecting drugs and toxicants in humans (Nosal et al. 2020, 2021). However, forensic analysis in cats remains in its early stages in Korea, because of the lack of standardized analysis methods and limited analytical case studies. In veterinary clinical settings, suspected toxicant exposure is often evaluated through indirect diagnostic methods (Yang et al. 2018).

However, these methods do not allow for the identification of the type or concentration of the toxic compound. Consequently, there is an urgent need to develop more precise and systematic analytical methods for diagnosing rodenticide poisoning in cats.

This study aimed to establish and validate an LC–MS/MS method for the detection of ARs in gastric samples from cats and to evaluate its utility when applied to veterinary forensic case samples.

This method offers advantages such as minimal solvent use and simplified sample handling. In this study, we present an LC–MS/MS method capable of simultaneously detecting eight ARs.

Instrumental analysis was performed using a quadrupole–linear ion trap (QTRAP) hybrid triple QTRAP mass spectrometer, which combined sensitive MRM acquisition with EPI scanning within a single analytical run. The establishment of this analytical method will enable early and accurate diagnosis of companion animal poisoning and is expected to enhance the reliability of toxicological evidence in veterinary forensic examinations.

## 2 Materials and Methods

### 2.1 Reagents and Chemicals

The analytical method targeted eight AR compounds: brodifacoum, bromadiolone, chlorophacinone, coumatetralyl, difenacoum, difethialone, flocoumafen and warfarin.

The standards, brodifacoum, bromadiolone, coumatetralyl, flocoumafen and warfarin, were purchased from Kemidas (Suwon, Korea). Chlorophacinone, difethialone and difenacoum were purchased from LGC Standards (Wesel, Germany).

Acetonitrile and methanol (MS grade) were purchased from Fisher Scientific Inc (Fair Lawn, NJ, USA). Ammonium acetate, acetic acid (≥ 99%) and formic acid (analytical grade, ≥ 98%) were purchased from Sigma‐Aldrich (St. Louis, MO, USA). Purified water was obtained using a Milli‐Q system (Millipore, Billerica, MA, USA).

ISOLUTE AOAC Waxed cSPE cartridges (350 mg/3 mL, Part No. Q0050‐0035‐BG; Biotage, Uppsala, Sweden) were used for automated sample clean‐up with a Biotage Extrahera automated sample preparation system. The cartridges contained primary–secondary amine (PSA), end‐capped C18 (C18EC) and anhydrous magnesium sulphate at a ratio of 1:1:3. Samples were filtered through 0.2‐µm polyvinylidene fluoride syringe filters (Whatman, Clifton, NJ, USA) before LC–MS/MS analysis.

### 2.2 Preparation of Standard Stock and Working Solutions

Five rodenticide standards (brodifacoum, bromadiolone, coumatetralyl, flocoumafen and warfarin) were custom‐prepared by Kemidas (Suwon, Korea) using methanol as the solvent to achieve a final concentration of 100 µg/mL each. Individual stock solutions of chlorophacinone, difethialone and difenacoum were prepared in methanol (1 mg/mL) and stored at −80°C.

Working solutions were generated by diluting the stock solutions with methanol to achieve concentrations in the range of 1–100 ng/mL. The working solutions were stored at 4°C. A separate set of stock solutions was diluted with methanol to prepare quality control (QC) solutions. Validation QC samples were prepared by spiking the gastric tissue with QC solutions, yielding final concentrations of 10, 20 and 50 µg/kg.

### 2.3 Necropsy and Sample Preparation for Analysis

This study included 747 cat forensic toxicology cases submitted to the Animal and Plant Quarantine Agency between January 2020 and August 2025. These cases involved cats for which toxicological examination was requested because poisoning was suspected based on the circumstances of discovery, submitted case information or clinical and gross pathological findings. Gastric tissue or gastric contents were collected from each case, and all 747 cases were analysed for the eight target ARs using the validated LC–MS/MS–QTRAP method.

Cat carcasses were transported to the Animal and Plant Quarantine Agency at the request of the police after death or upon discovery. Necropsies were performed immediately after thawing.

Following necropsy, representative gastric contents or tissue were aseptically collected from each animal under controlled conditions to prevent cross‐contamination. The samples were immediately frozen at –20°C to preserve analyte stability and stored until further analysis. Ethical approval was not required because the study used only post‐mortem samples collected during routine veterinary forensic examinations and involved no procedures on live animals.

Sample preparation was performed using a modified AOAC QuEChERS (Quick, Easy, Cheap, Effective, Rugged and Safe) method. This method combines acetonitrile extraction with cartridge‐based solid‐phase extraction clean‐up to efficiently extract the target analytes and remove matrix‐derived interferences from complex biological matrices, such as gastric contents or gastric tissue.

Peres et al.’s (2019) method was consulted and selectively revised and optimized for our study objectives.

Approximately 1.00 ± 0.05 g of homogenized gastric content was accurately weighed and transferred into a 50 mL centrifuge tube, followed by the addition of 3 mL of acetonitrile containing 1% acetic acid. The mixture was vigorously shaken for 10 min to ensure efficient extraction of the target analytes and then centrifuged at 4000 × *g* for 10 min to separate the upper organic phase.

A 3 mL aliquot of the resulting supernatant was automatically loaded onto an ISOLUTE AOAC Waxed 350 mg/3 mL cartridge (Part No. Q0050‐0035‐BG; Biotage) using the Biotage Extrahera automated sample preparation system. Interfering substances present in the samples were selectively adsorbed onto the sorbents within the cartridge, including PSA and C18EC.

The target analytes were subsequently eluted into glass test tubes through the automated sample preparation system. The collected eluate was completely evaporated under a stream of nitrogen, reconstituted in 1 mL of methanol, and filtered through a 0.2 µm polyvinylidene fluoride syringe filter prior to LC–MS/MS analysis.

### 2.4 LC–MS/MS Method

Chromatographic separation was conducted using an LC‐20AD (SHIMADZU, Kyoto, Japan) equipped with a YMC‐Triart C18 column (100 mm × 3 mm, 3 µm) (YMC, Torrance, CA, USA) maintained at a temperature of 40°C. The mobile phase consisted of (A) 5 mM ammonium acetate and 0.1% formic acid in distilled water and (B) methanol containing 0.1% formic acid, delivered at a flow rate of 300 µL/min. The initial mobile phase composition (10% B, v/v) was held for 0.25 min, followed by a linear gradient increase from 10% to 90% B over 7.75 min. The composition was maintained at 90% B until 13.0 min, then decreased to 10% B at 13.1 min, and re‐equilibrated at 10% B until 15 min. The total runtime for the method was 15 min, with an injection volume of 2 µL.

Mass spectrometry analysis was performed using an AB SCIEX QTRAP 5500+ mass spectrometer (AB SCIEX Instruments, Foster, CA, USA) with an ESI interface. Data were acquired in MRM mode using negative ESI. The mass spectrometry conditions were as follows: ion spray voltage, –4500 V; curtain gas pressure, 40 psi; ion source gas 1: 50 psi; ion source gas 2: 50 psi; and source temperature, 500°C.

To minimize false positives and enhance identification confidence in multi‐analyte screening for veterinary forensic examinations, we employed information‐dependent acquisition (IDA) for qualitative data collection. Precursor ions exceeding 500 counts/s^−1^ in the real‐time survey MS1 scan were selected to acquire EPI spectra under the following conditions: m/z 50–600 and a scan rate of 10,000 Da/s^−1^. The resulting spectra were evaluated for structural concordance of fragment ions, thereby increasing the confidence in compound identification.

### 2.5 Validation of LC–MS/MS Method

The LC–MS/MS method was comprehensively validated for sensitivity, linearity, precision, accuracy, limit of detection and limit of quantification for eight compounds.

Linearity was evaluated by constructing a calibration curve using six different concentration points ranging from 1 to 100 ng/mL. The coefficient of determination (*r* ^2^) was calculated to determine the degree of linearity and ensure reliability of the calibration curve.

To verify the accuracy of the method, a recovery test was conducted by spiking a blank sample with known amounts of the target analyte to achieve final concentrations of 10, 20 and 50 µg/kg. The spiked samples were then analysed, and recovery rates were calculated to verify that the method provides accurate and reproducible results.

Precision was assessed using replicate measurements under the same conditions and calculating the RSD. This evaluation confirmed that the method provides consistent results in repeated analyses.

The detection capability of the method was evaluated by determining the LOD and LOQ. The LOD was defined as the lowest concentration at which the analyte could be reliably detected with a signal‐to‐noise (S/N) ratio ≥ 3, whereas the LOQ was defined as the lowest concentration that could be accurately quantified with an S/N ratio ≥ 10. These criteria were established to assess the suitability of the method for the detection and quantification of target compounds at trace levels.

An acceptable retention‐time tolerance of ±0.1 min relative to the reference standard was applied, and the ion‐ratio criterion was set to ±20% of the calibration‐standard reference values.

## 3 Results

### 3.1 Optimization of the LC–MS/MS Method

Full‐scan and MS/MS spectra for all rodenticides were acquired by injecting individual standard solutions prepared in methanol into the mass spectrometer. Each rodenticide was evaluated in both positive and negative electrospray ionization modes. The precursor/product ions, retention times and optimized MS parameters (DP, CE, CEP and CXP) are summarized in Table 1. For most rodenticides, the ESI‐mode provided superior sensitivity.

**TABLE 1:** Optimized multiple reaction monitoring and mass spectrometry parameters for eight ARs.

Compound	Formula	RT (min)	Q1 Mass(Da)	Q3 Mass(Da)	DP (volt)	EP (volt)	CE (volt)	CXP (volt)
Brodifacoum	C_31_H_23_BrO_3_	11.05	521.1 [M+H]^−^	79.1	−60	−10	−95	−9
				135.1	−60	−10	−50	−8
Bromadiolone	C_30_H_23_BrO_4_	9.87	525.1 [M+H]^−^	250.0	−60	−10	−55	−14
				79.1	−60	−10	−95	−12
Coumatetralyl	C_19_H_16_O_3_	9.26	291.1 [M+H]^−^	141.0	−60	−10	−40	−11
				247.2	−60	−10	−30	−10
Chlorophacinone	C_23_H_15_ClO_3_	10.31	373.1 [M+H]^−^	201.1	−60	−10	−30	−11
				145.1	−60	−10	−30	−12
Difenacoum	C_31_H_24_O_3_	10.60	443.2 [M+H]^−^	293.2	−60	−10	−45	−8
				135.1	−60	−10	−50	−7
Difethialone	C_31_H_23_BrO_2_S	11.61	537.1 [M+H]^−^	79.1	−60	−10	−80	−12
				81.1	−60	−10	−80	−12
Flocoumafen	C_33_H_25_F_3_O_4_	10.42	541.2 [M+H]^−^	161.2	−60	−10	−45	−10
				382.1	−60	−10	−30	−10
Warfarin	C_19_H_16_O_4_	8.79	307.1 [M+H]^−^	250.1	−60	−10	−30	−9
				161.1	−60	−10	−35	−10

Abbreviations: CE, collision energies; CXP, collision cell exit potential; DP, declustering potentials; EP, entrance potentials; RT, retention time.

**FIGURE 1:**
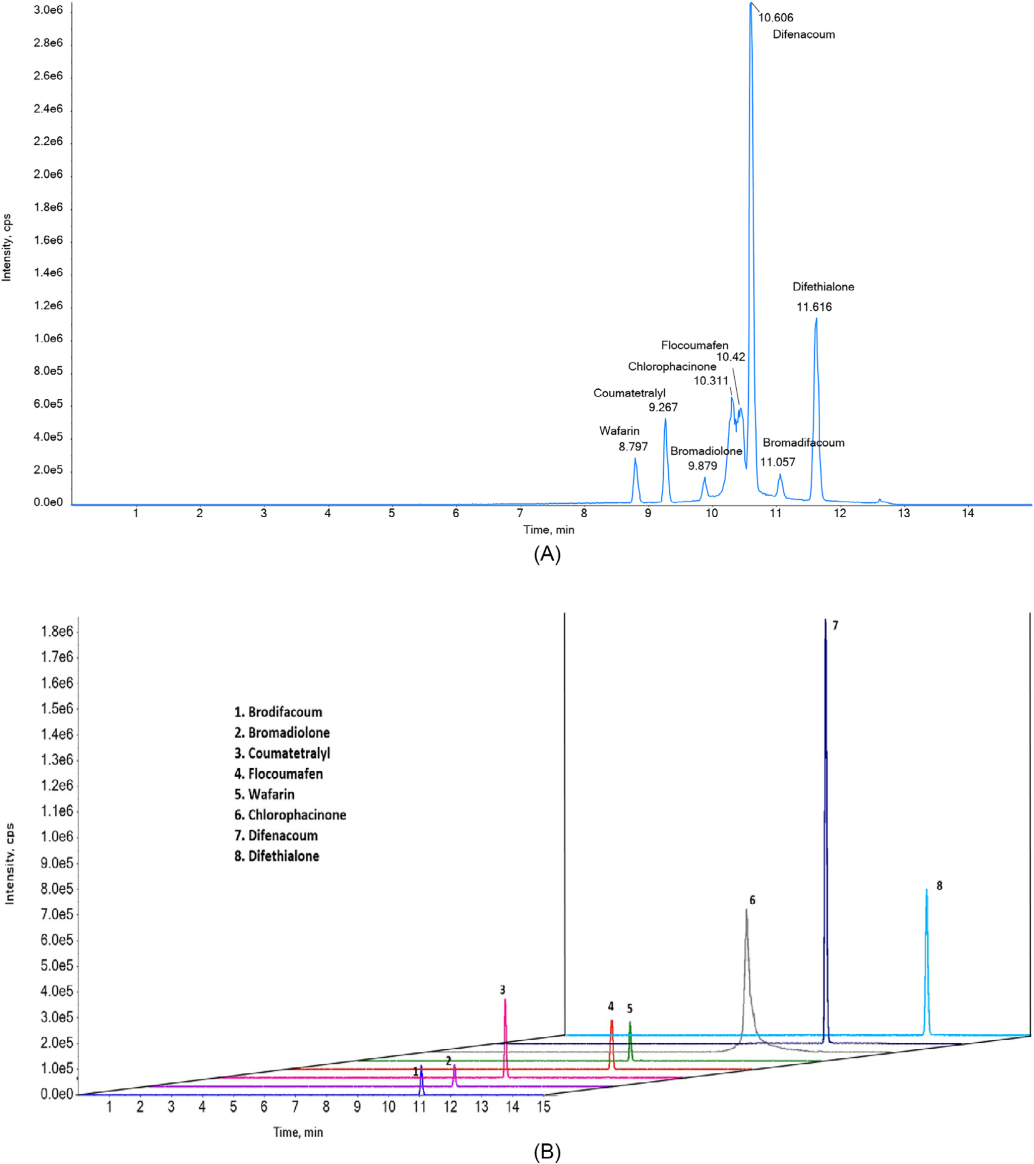
(A) Total ion chromatograms and (B) extracted ion chromatograms of the eight rodenticides added to blank gastric tissue.

Each compound was characterized by its retention time and two product ions. In accordance with the 2002/657/EC guidelines (European Commission 2002), the most abundant product ion was selected as the quantifier ion, whereas the second product ion was used as the qualifier ion. Figure 1A shows the total ion chromatograms (TIC) of eight rodenticides spiked into blank gastric tissue and analysed by LC–MS/MS. As shown in the extracted ion chromatograms, all target rodenticides were well separated within 15 min (Figure 1B).

### 3.2 Method Validation

In this study, an optimized LC–MS/MS‐based analytical method was validated according to the guidelines for the quantification of eight anticoagulants in the gastric tissues of cats. This method was validated to assess its reliability and applicability in veterinary forensic medicine. The validation parameters included selectivity, linearity, LOD, LOQ, accuracy and precision.

Selectivity was evaluated to determine whether endogenous substances or structurally related compounds interfered with the detection of the target analytes. Five blank gastric tissue samples from cats without anticoagulant exposure were analysed. No interfering peaks were observed in the retention times of the target analytes.

Linearity was assessed by constructing calibration curves at six concentrations (1, 5, 10, 20, 50 and 100 ng/mL), each analysed in triplicate. Least‐squares regression with 1/*x* weighting was used. The coefficients of determination (*r* ^2^) for all the analytes ranged from 0.990 to 0.997, demonstrating excellent linearity within the tested concentration range.

The LOD and LOQ were defined based on S/N ratios of ≥ 3 and ≥ 10, respectively. The LOD values were in the range of 0.05–0.1 µg/kg, and the LOQ values were between 0.1 and 0.5 µg/kg, indicating that the method had sufficient sensitivity to reliably detect even trace levels of anticoagulants in gastric tissue.

Accuracy was evaluated using QC samples prepared by spiking blank gastric tissue samples with the target compounds at three concentrations (10, 20 and 50 µg/kg). Each concentration was analysed six times. The accuracy, calculated as the ratio of measured to spiked concentrations, ranged from 70.3% to 105.6%, meeting the internationally accepted criterion of 70%–120%.

Precision was assessed by calculating the RSD (%) of repeated measurements. RSD values for all analytes ranged from 2.4% to 8.6%, satisfying the acceptance criteria of ≤ 15%, and demonstrating the high repeatability of the method (Table 2 **)**.

**TABLE 2:** Analytical performance including calibration (*r* ^2^), recovery, repeatability (RSD), LOD and LOQ for eight ARs.

Compound	*r* ^2^	Recovery (%)	RSD (%)	LOD (µg/kg)	LOQ (µg/kg)
Brodifacoum	0.996	74.3–95.7	7.7–8.6	0.1	0.5
Bromadiolone	0.994	71.9–98.6	3.8–7.1	0.1	0.5
Coumatetralyl	0.995	86.5–102.3	4.0–5.7	0.05	0.1
Chlorophacinone	0.992	82.3–91.8	5.6–8.1	0.1	0.5
Difenacoum	0.991	70.3–93.2	5.1–8.3	0.1	0.5
Difethialone	0.990	73.6–103.7	6.9–7.1	0.1	0.5
Flocoumafen	0.993	71.6–96.5	4.7–7.3	0.1	0.5
Warfarin	0.997	80.3–105.6	2.4–6.7	0.05	0.1

Abbreviations: LOD: Limit of detection; LOQ: Limit of quantitation; RSD: Relative standard deviation.

*r* ^2^, Coefficient of determination.

Taken together, the validated LC–MS/MS method fulfilled all internationally accepted criteria for analytical performance, including selectivity, linearity, sensitivity, accuracy and precision. Therefore, the validated method is suitable for the reliable detection and quantification of ARs in cat gastric tissue.

### 3.3 Monitoring of AR Poisoning in Cats (2020–2025)

Between 2020 and August 2025, our laboratory received a total of 747 requests for toxicological analysis of cat necropsy samples, including 67 in 2020, 173 in 2021, 243 in 2022, 95 in 2023, 108 in 2024 and 61 in 2025. All 747 cases were analysed for the eight target ARs using the validated LC–MS/MS‐QTRAP method. ARs were detected in 19 cases, corresponding to an overall detection rate of 2.5% (19/747).

Among the 19 AR‐positive cases, coumatetralyl alone was detected in 14 cases (73.7%), flocoumafen alone in 3 cases (15.8%), both coumatetralyl and flocoumafen in 1 case (5.3%) and bromadiolone alone in 1 case (5.3%) (Table 3). Co‐detection occurred in one case (Animal ID: 2023Q174), in which coumatetralyl (79.1 µg/kg) and flocoumafen (50.5 µg/kg) were detected in the gastric contents from one specimen. Coumatetralyl concentrations ranged from 2.3 to 107.6 µg/kg (mean, 42.6 µg/kg; *n* = 15), whereas flocoumafen concentrations ranged from 15.9 to 80.3 µg/kg (mean, 43.6 µg/kg; *n* = 4).

**TABLE 3:** Overview of AR detections of coumatetralyl flocoumafen in companion animal necropsy cases (January 2020–August 2025).

Animal ID	Matrix	Detected compounds	Conc. (µg/kg)	Necropsy requester	Carcass conservation	Related findings
2020Q057	Gastric contents	Coumatetralyl	97.3	Police	Frozen	Severe pulmonary haemorrhage
2021Q081	Gastric tissue	Coumatetralyl	16.5	Police	Frozen	Signs of bleeding around the nose and mouth.
2021Q194	Gastric contents	Bromadiolone	43.4	Police	Frozen	Severe pulmonary haemorrhage
2021Q246	Gastric tissue	Coumatetralyl	12.9	Police	Frozen	Intraluminal pooling of haemorrhagic fluid
2022Q007	Gastric contents	Coumatetralyl	31.6	Police	Frozen	Jaundice and petechial haemorrhages
2022Q049	Gastric tissue	Coumatetralyl	10.8	Police	Frozen	Signs of bleeding around the nose and mouth.
2022Q083	Gastric tissue	Flocoumafen	27.8	Police	Frozen	Intraluminal pooling of haemorrhagic fluid
2022Q126	Gastric tissue	Coumatetralyl	18.2	Police	Frozen	Haemorrhagic lesions around the mesentery
2022Q185	Gastric contents	Flocoumafen	80.3	Police	Frozen	Severe pulmonary hyperaemia
2022Q186	Gastric tissue	Flocoumafen	15.9	Police	Frozen	Signs of bleeding around the nose and mouth.
2022Q213	Gastric contents	Coumatetralyl	95.8	Police	Frozen	Severe pulmonary haemorrhage
2023Q016	Gastric tissue	Coumatetralyl	2.3	Municipal animal shelter	Frozen	Signs of bleeding around the nose and mouth.
2023Q033	Gastric tissue	Coumatetralyl	24.5	Police	Frozen	Signs of bleeding around the nose and mouth.
2023Q106	Gastric contents	Coumatetralyl	33.6	Owner	None	Severe pulmonary hyperaemia
2023Q107	Gastric contents	Coumatetralyl	107.6	Police	Frozen	Severe pulmonary hyperaemia
2023Q174	Gastric contents	Coumatetralyl	79.1	Police	Refrigerated	Jaundice and petechial haemorrhages
		Flocoumafen	50.5			
2023Q388	Gastric contents	Coumatetralyl	43.3	Police	Frozen	Severe pulmonary haemorrhage
2024Q063	Gastric tissue	Coumatetralyl	37.9	Police	Frozen	Signs of bleeding around the nose and mouth.
2025Q075	Gastric tissue	Coumatetralyl	28.3	Police	Frozen	Intraluminal accumulation of blood‐tinged fluid

Among the AR‐positive forensic cases, specimen types were similarly distributed between gastric tissue (10/19, 52.6%) and gastric contents (9/19, 47.4%). Most submissions originated from law enforcement (17/19, 89.5%), with one each from a municipal shelter and an owner. Carcasses were predominantly submitted frozen (17/19, 89.5%), with one refrigerated and one unpreserved.

Information on the geographic origin of the AR‐positive cases was not consistently available in the submitted case records. Therefore, the regional distribution of AR exposure could not be evaluated in this study. Among the nine AR‐positive cases in which gastric contents were available, no recognizable rodenticide bait material or prey remains were identified on gross examination. Therefore, the source of AR exposure and the distinction between primary and secondary exposure could not be determined from the gastric‐content findings.

The annual distribution of AR‐positive cases was as follows: one case in 2020, three in 2021, seven in 2022, six in 2023, one in 2024 and one in 2025. Haemorrhagic findings were observed in several AR‐positive cases.

The most frequent findings were perinasal/perioral bleeding (6/19, 31.6%), followed by extensive pulmonary haemorrhage (4/19, 21.1%) and pulmonary congestion/hyperaemia (3/19,15.8%). Additional observations included blood‐tinged gastrointestinal contents (2/19, 10.5%), jaundice with petechiae (2/19, 10.5%), haemorrhagic lesions around the mesentery (1/19, 5.3%) and generalized accumulation of blood‐tinged fluid (1/19, 5.3%). Although these lesions are frequently reported in association with AR poisoning, they are not specific and may also occur with trauma, primary coagulopathies, disseminated intravascular coagulation, infectious diseases and other systemic conditions. The observed haemorrhagic lesions were consistent with the known anticoagulant effects of ARs, which inhibit vitamin K epoxide reductase and reduce the activity of clotting factors II, VII, IX and X (Mount 1988). When considered together with the analytical detection of coumatetralyl, flocoumafen, or bromadiolone, these findings were compatible with AR‐associated coagulopathy; however, the contribution of AR exposure to death could not be definitively established in all cases.

Overall, coumatetralyl was the predominant AR detected, followed by flocoumafen and bromadiolone.

**FIGURE 2:**
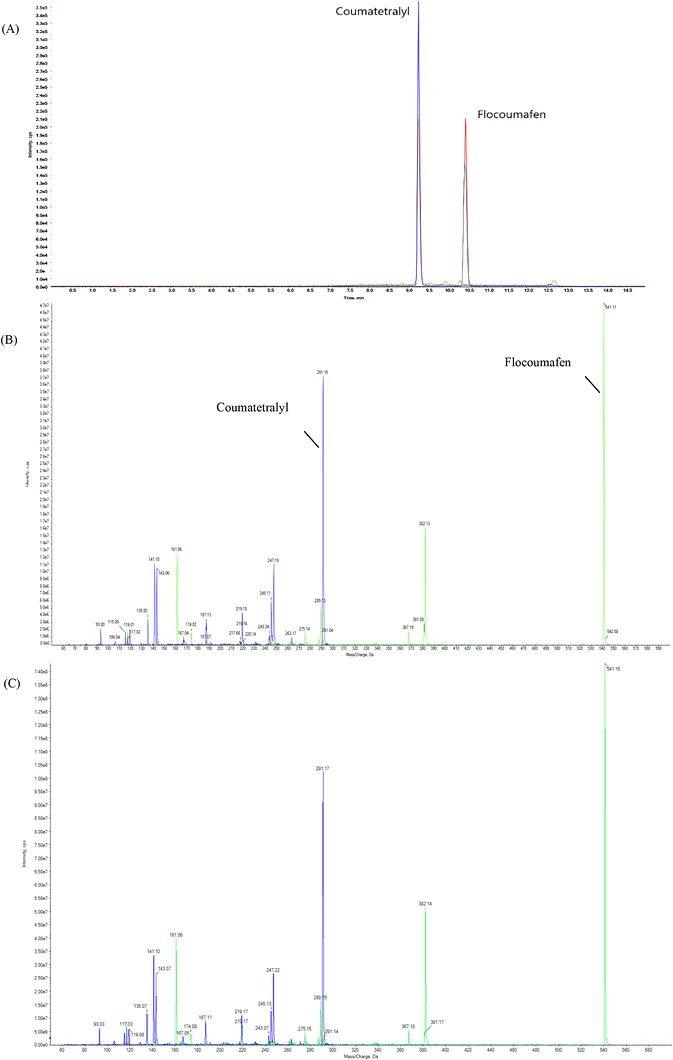
LC–MS/MS confirmation of coumatetralyl and flocoumafen in a cat poisoning case (2023Q174). (A) Extracted ion chromatograms showing the co‐detection of coumatetralyl and flocoumafen in the sample. (B) EPI spectra of reference standards for coumatetralyl and flocoumafen. (C) Corresponding EPI spectra acquired from the sample for coumatetralyl and flocoumafen.

All AR‐positive samples were further confirmed using a secondary QTRAP‐based identification procedure. Specifically, EPI spectra acquired through IDA were compared with those of the corresponding reference standards to confirm spectral concordance. EPI spectra matched in all samples, minimizing the possibility of false positives due to structural analogues. Representative chromatograms and EPI spectra of coumatetralyl and flocoumafen are presented in Figure 2.

## 4 Discussion

This study established a standardized LC–MS/MS‐QTRAP analytical method combined with automated SPE‐based sample preparation for the rapid and accurate identification of eight ARs in gastric matrices from cats, including gastric tissue and gastric contents. The effectiveness of this method was demonstrated by applying it to 747 cat forensic toxicology cases submitted between January 2020 and August 2025. In addition, our laboratory successfully completed the proficiency test (Test No.: KT 1025) and was accredited as an internationally recognized testing laboratory by the Korea Laboratory Accreditation Scheme (KOLAS) in October 2025 for ISO/IEC 17025.

Coagulation tests such as PT and aPTT, which are widely used in routine clinical practice, have inherent limitations in that they can be nonspecifically prolonged in various pathological conditions, such as liver disease, inflammation and malnutrition. These tests may also show values within reference ranges during the early stages of exposure, making it difficult to identify the presence and type of toxic substance (Waddell et al. 2013; Conroy et al. 2024). The approach used in this study addresses some of these limitations. By combining Biotage Extrahera‐based automated solid‐phase extraction with AOAC Waxed cartridges, multiple matrix‐derived interferences were effectively reduced while ensuring consistent recovery and repeatability. A standardized sample‐preparation procedure was also implemented to minimize inter‐analyst variability. Furthermore, the LC–MS/MS conditions established in this study enabled simultaneous separation and quantification of eight ARs within a total runtime of 15 min. This method is significant because it goes beyond determining exposure and allows rapid compound‐specific identification at the molecular level, thereby refining veterinary forensic judgments regarding the type of toxicant, possible mixed exposure.

In practical application, all 747 submitted cat veterinary forensic toxicology cases were analysed for the eight target ARs using the validated LC–MS/MS‐QTRAP method. ARs were detected in 19 of these 747 cases, corresponding to an AR‐positive rate of 2.5%. The 747 cases refer to cases in which toxicological testing was specifically requested and performed; they do not represent all cat necropsy cases received during the study period. Because these submissions included cats with various suspected causes of death, the proportion of AR‐positive cases was relatively low. Accordingly, the observed detection frequency should be interpreted within the scope of the submitted toxicology cases analysed in this study. Dedicated surveillance data or market‐based studies would be required to properly evaluate broader trends, including national AR exposure prevalence and rodenticide product use patterns.

Coumatetralyl was the most frequently detected AR, occurring in 15 of the 19 AR‐positive cases. Coumatetralyl and flocoumafen were detected simultaneously in one case, suggesting possible exposure to mixed products or multiple AR sources and supporting the need for simultaneous multi‐analyte analysis in veterinary forensic examinations. This coumatetralyl‐dominant pattern may, at least in part, reflect domestic exposure patterns in South Korea, where rodenticide products are managed under a national registration system.

The detection of flocoumafen is of particular significance because it was not listed as a registered active ingredient in the domestic rodenticide registration information available for the study period. Rodenticide registration and management in South Korea are overseen by the National Institute of Environmental Research. Based on the domestic registration records reviewed for the study period, registered rodenticide formulations in South Korea were based primarily on coumatetralyl and bromadiolone. The predominance of coumatetralyl among the AR‐positive cases may therefore partly reflect the availability of coumatetralyl‐containing products in South Korea.

In contrast, the detection of flocoumafen suggests that cats may have been exposed to products or sources not represented in the available domestic registration records. However, comprehensive information on product availability, formulation types and distribution routes was not available, and the specific source of flocoumafen exposure could not be determined from the present case.

These findings indicate that veterinary forensic examination should not be restricted to active ingredients documented in domestic registration records and support broad multi‐analyte screening that includes both registered and potentially unregistered ARs. Previous studies on AR detection have primarily focused on liver tissue or blood (Chalermchaikit et al. 1993; Francis et al. 2023). Liver is often a key matrix for diagnosing AR exposure, particularly when the post‐ingestion interval is prolonged, repeated exposure is suspected or tissue persistence is expected. This is especially relevant for second‐generation ARs, which have longer biological persistence and may accumulate in tissues. However, in routine cat forensic necropsy cases, sample availability is frequently limited due to small body size, post‐mortem condition and the need to allocate tissues for essential examinations, such as bacteriology and histopathological analyses. Because AR intoxication in cats is commonly associated with oral exposure, gastric tissue and gastric contents were prioritized as practical matrices and the method was validated accordingly.

The use of gastric tissue and gastric contents is particularly valuable for evaluating recent oral exposure. These matrices are closely associated with the ingestion of contaminated food, bait or prey and may allow the detection of AR residues before extensive distribution and metabolism occur in the body. Therefore, gastric samples may provide useful evidence supporting recent ingestion. However, these matrices cannot by themselves distinguish between primary and secondary exposure or determine whether exposure was accidental or deliberate. In the present study, no recognizable rodenticide bait material or prey remains were identified on gross examination of the nine AR‐positive gastric‐content samples. Although the absence of such material does not exclude either direct bait ingestion or secondary exposure through contaminated prey, it did not permit definitive determination of the exposure pathway. Therefore, neither the exposure pathway nor the intentionality of exposure could be determined, and the toxicological findings should be interpreted together with broader veterinary forensic, environmental and investigative information.

Cats may be exposed to ARs through both primary and secondary pathways. Primary exposure may occur through direct ingestion of rodenticide bait, contaminated food or bait‐like materials placed in areas accessible to cats. Secondary exposure may occur through ingestion of rodents or other prey that have previously consumed AR bait (Cerkvenik‐Flajs et al. 2025; Mahjoub et al. 2022). This is particularly relevant because cats are predatory animals and may encounter rodents in urban, peri‐urban or agricultural environments. Keating et al. (2024) reviewed AR exposure in mammalian carnivores and emphasized that these animals can be exposed through contaminated prey, supporting the need to consider both direct bait ingestion and secondary consumption when interpreting AR residues in cats. Therefore, both primary and secondary exposure pathways should be considered when evaluating AR‐positive cat forensic cases.

Compared with previous studies involving cats, Kohn et al. (2003) reported haemorrhagic manifestations in seven cats with suspected AR intoxication, demonstrating that AR exposure may be associated with clinically significant coagulopathy in cats. In contrast, Mahjoub et al. (2022) reported asymptomatic AR exposure in dogs and cats, indicating that exposure may occur in the absence of overt clinical signs. More recently, Cerkvenik‐Flajs et al. (2025) documented rodenticide exposure in domestic cats, further supporting the occurrence of AR exposure under field conditions. Unlike these clinical or tissue residue studies, the present study examined veterinary forensic cases using gastric tissue and gastric contents as the principal analytical matrices. Therefore, the present findings primarily support recent oral exposure rather than long‐term tissue persistence, cumulative exposure or exposure prevalence in the general cat population.

The findings of this study are consistent with previous reports showing that AR exposure can occur in non‐target animals, including cats and wildlife (Sánchez‐Barbudo et al. 2012). Sánchez‐Barbudo et al. (2012) reported AR residues in a wide range of non‐target animals in Spain and discussed both primary and secondary poisoning pathways. Compared with that liver‐based survey, the present study differs by focusing specifically on cat gastric tissue and gastric contents in forensic casework. This difference in matrix selection is important because liver residues are more informative for persistence, bioaccumulation and repeated exposure, whereas gastric contents and gastric tissue are more directly related to recent oral ingestion. López‐Perea et al. (2019) also reported AR exposure in non‐target animals and emphasized the influence of anthropogenic and environmental factors on exposure risk, supporting the importance of considering habitat, rodenticide use patterns and predator–prey pathways when interpreting AR residues. Previous veterinary toxicology findings have shown that gastric contents may remain positive even when liver samples are negative, supporting their complementary value in suspected pesticide and rodenticide poisoning investigations (Lahmar et al. 2019).

Consequently, the application and validation of this method to gastric matrices from cats expands upon previous liver‐ and blood‐focused studies and strengthens evidence for recent oral exposure in veterinary forensic examination.

The findings of the present study are more consistent with recent oral AR exposure than with repeated or prolonged exposure. Haemorrhagic findings in AR‐positive cats, including pulmonary haemorrhage, perinasal or perioral bleeding and blood‐tinged gastrointestinal contents, were compatible with AR‐associated coagulopathy (Mount 1988; Kohn et al. 2003). These findings are consistent with the known mechanism of AR poisoning, in which inhibition of vitamin K epoxide reductase reduces the activity of vitamin K‐dependent clotting factors II, VII, IX and X (Mount 1988).

The concurrence of analytical AR detection and haemorrhagic lesions suggests that AR exposure may have contributed to death in some cases; however, a causal relationship could not be established because the lesions were nonspecific and complete clinicopathological and investigative information was not consistently available.

Because gastric tissue and gastric contents were the principal matrices analysed, the positive findings primarily indicate recent ingestion. Gastric contents may retain unabsorbed AR residues at the time of death and may therefore provide complementary evidence of recent oral exposure (Chansiripornchai et al. 2025). In contrast, clinical AR intoxication in cats has been associated with anaemia, lethargy, anorexia and respiratory abnormalities (Kohn et al. 2003). Repeated or prolonged exposure may contribute to generalized weakness or reduced activity, potentially increasing an animal's vulnerability to environmental hazards or other adverse outcomes. However, direct evidence that repeated low‐level AR exposure causes impaired mobility, behavioural changes, or indirect mortality in cats remains limited (Kopanke et al. 2018).

Liver tissue is more suitable for evaluating previous or persistent exposure because some ARs, particularly second‐generation compounds, have prolonged hepatic elimination and may remain detectable for extended periods (Vandenbroucke, Bousquet‐Mélou, et al. 2008). Therefore, the lack of consistent liver analysis in the present study limited the assessment of previous low‐level or repeated exposure.

LC–MS/MS is widely used in the field of rodenticide analysis, and this technique offers high selectivity and excellent sensitivity (Bertolini et al. 2024; Arens et al. 2022). In particular, the liquid chromatography–triple QTRAP method can simultaneously acquire quantitative MRM and qualitative QTRAP scans, such as EPI, in a single run, providing richer spectral information and improving the identification accuracy of target analytes compared with conventional MS/MS approaches (Mueller et al. 2005; Heffernan et al. 2016). The LC–MS/MS‐QTRAP method in this study maintained the high sensitivity of MRM while simultaneously performing EPI spectral‐based confirmation, thereby reducing the risk of false‐positive identification due to structural analogues. The dual‐confirmation approach consisting of MRM screening and EPI structural verification improves identification reliability even in complex matrices. This method is useful for veterinary forensic toxicology, particularly for the interpretation of suspected AR exposure in cats, and may contribute to toxicovigilance by enabling reliable multi‐analyte detection with MRM/EPI confirmation in gastric matrices from cats.

Taken together, this study demonstrates the forensic utility of LC–MS/MS‐QTRAP analysis of gastric matrices from cats for identifying recent AR exposure in suspected poisoning cases. The detection of both registered and unregistered ARs highlights the need for comprehensive multi‐analyte screening and continued toxicovigilance to better characterize AR exposure patterns and associated risks in cats and other non‐target animals.

## 5 Conclusion

This study developed and validated an LC–MS/MS‐QTRAP method for the simultaneous detection and quantification of eight ARs in gastric matrices from cats. The optimized QuEChERS sample preparation reduced matrix‐related interferences and provided a practical approach for analysing gastric tissue and gastric contents from forensic cat cases. By combining MRM quantification with IDA‐EPI spectral confirmation, the method enhanced confidence in AR identification in complex biological samples. The 15‐min analytical runtime further indicates its applicability to routine veterinary forensic toxicology. These findings indicate that gastric matrices from cats can provide useful evidence of AR exposure and that this method may contribute to improved investigation and toxicovigilance of suspected rodenticide poisoning in cats.

## Author Contributions

**HyunYoung Chae**: conceptualization, data curation, formal analysis, investigation, methodology, software, validation, visualization, writing – original draft, writing – review and editing. **Jae‐Won Byun**: investigation, project administration, resources, supervision. **So‐Jeong Yim**: formal analysis. **Bok‐Kyung Ku**: funding acquisition, project administration, resources, supervision. **Tae‐Wan Kim**: investigation, writing – review and editing. **JeongWoo Kang**: conceptualization, funding acquisition, investigation, methodology, project administration, resources.

## Funding

This study was supported by veterinary science research project grants from the Korean Animal and Plant Quarantine Agency (B‐1543069‐2025‐26‐01).

## Conflicts of Interest

The authors declare no conflicts of interest.

## Data Availability

The data that support the findings of this study are available from the corresponding author upon reasonable request.

## References

[vms371209-bib-0001] American Society for the Prevention of Cruelty to Animals . 2025. “Rodenticide and Your Pet: What You Need to Know.” ASPCA. Published November 11. https://www.aspca.org/news/rodenticide‐and‐your‐pet‐what‐you‐need‐know.

[vms371209-bib-0002] Arens, A. , J. Teske , M. Klintschar , R. Mischke , and M. Dziadosz . 2022. “Antemortem and Postmortem Rodenticide Analysis in Forensic Toxicology as a Part of an LC–MS/MS‐Based Multi‐Target Screening Strategy.” Drug Testing and Analysis 14: 1149–1154. 10.1002/dta.3222. 34997698

[vms371209-bib-0003] ASPCA Animal Poison Control Center . 2024. “Top 10 Pet Toxins of 2023.” ASPCA PRO. Accessed June 22, 2026. https://www.aspcapro.org/resource/top‐10‐pet‐toxins‐2023.

[vms371209-bib-0004] ASPCA Animal Poison Control Center . 2025. “Top 10 Toxins of 2024.” ASPCA PRO. Accessed June 22, 2026. https://www.aspcapro.org/resource/top‐10‐toxins‐2024.

[vms371209-bib-0005] Berny, P. J. , T. Buronfosse , and G. Lorgue . 1995. “Anticoagulant Poisoning in Animals: A Simple New High‐Performance Thin‐Layer Chromatographic (HPTLC) Method for the Simultaneous Determination of Eight Anticoagulant Rodenticides in Liver Samples.” Journal of Analytical Toxicology 19, no. 7: 576–580. 10.1093/jat/19.7.576. 8577181

[vms371209-bib-0006] Bertolini, F. M. , E. Barolo , R. Masti , et al. 2024. “Fast and Sensitive Method for the Diagnosis and Follow‐Up of Anticoagulant Rodenticides Poisoning in Animal Whole Blood.” Journal of Chromatography B 1232: 123971. 10.1016/j.jchromb.2023.123971. 38128166

[vms371209-bib-0007] Bille, L. , M. Toson , P. Mulatti , et al. 2016. “Epidemiology of Animal Poisoning: An Overview on the Features and Spatio‐Temporal Distribution of the Phenomenon in the North‐Eastern Italian Regions.” Forensic Science International 266: 440–448. 10.1016/j.forsciint.2016.07.002. 27450041

[vms371209-bib-0008] Cerkvenik‐Flajs, V. , D. Schenke , S. Korenjak‐Černe , et al. 2025. “Exposure of Domestic Cats (*Felis catus*) to Rodenticidal Compounds.” Toxics 13, no. 8: 663. 10.3390/toxics13080663. 40863939 PMC12390110

[vms371209-bib-0009] Chalermchaikit, T. , L. J. Felice , and M. J. Murphy . 1993. “Simultaneous Determination of Eight Anticoagulant Rodenticides in Blood Serum and Liver.” Journal of Analytical Toxicology 17, no. 1: 56–61. 10.1093/jat/17.1.56. 8429630

[vms371209-bib-0010] Chansiripornchai, P. , V. Hunprasit , and S. Techangamsuwan . 2025. “First Report on the Occurrence of Anticoagulant Rodenticides Toxicosis in Nontarget Animals in Thailand.” BMC Veterinary Research 21: 337. 10.1186/s12917-025-04789-7. 40350410 PMC12066049

[vms371209-bib-0011] Conroy, E. M. , B. M. Lyons , and A. Koenig . 2024. “Evaluation of a Whole Blood Point‐of‐Care Coagulation Analyzer in Dogs.” Journal of Veterinary Emergency and Critical Care 34, no. 5: 446–454. 10.1111/vec.13416. 39226146

[vms371209-bib-0012] European Commission . 2002. “Commission Decision 2002/657/EC Implementing Council Directive 96/23/EC Concerning the Performance of Analytical Methods and the Interpretation of Results.” Official Journal of the European Communities 221: 8–36. https://eur‐lex.europa.eu/legal‐content/EN/TXT/?uri=CELEX:32002D0657.

[vms371209-bib-0013] Francis, K. A. , A. Tkachenko , J. T. Johnson , et al. 2023. “Comprehensive Evaluation of an HPLC–MS/MS Method for Quantitation of Seven Anti‐Coagulant Rodenticides and Dicoumarol in Animal Serum.” Journal of Analytical Toxicology 47, no. 5: 429–435. 10.1093/jat/bkad017. 36869712

[vms371209-bib-0014] Gallocchio, F. , L. Basilicata , C. Benetti , R. Angeletti , and G. Binato . 2014. “Multi‐Residue Determination of Eleven Anticoagulant Rodenticides by High‐Performance Liquid Chromatography With Diode Array/Fluorimetric Detection: Investigation of Suspected Animal Poisoning in the Period 2012–2013 in North‐Eastern Italy.” Forensic Science International 244: 63–69. 10.1016/j.forsciint.2014.08.012. 25195128

[vms371209-bib-0015] Griggs, A. N. , R. A. Allbaugh , K. L. Tofflemire , G. Ben‐Shlomo , D. Whitley , and M. E. Paulsen . 2016. “Anticoagulant Rodenticide Toxicity in Six Dogs Presenting for Ocular Disease.” Veterinary Ophthalmology 19, no. 1: 73–80. 10.1111/vop.12267. 25800104

[vms371209-bib-0016] Heestermans, M. , G. Poenou , H. Hamzeh‐Cognasse , F. Cognasse , and L. Bertoletti . 2022. “Anticoagulants: A Short History, Their Mechanism of Action, Pharmacology, and Indications.” Cells 11, no. 20: 3214. 10.3390/cells11203214. 36291080 PMC9600347

[vms371209-bib-0017] Heffernan, A. L. , K. Thompson , G. Eaglesham , et al. 2016. “Rapid, Automated Online SPE–LC–QTRAP–MS/MS Method for the Simultaneous Analysis of 14 Phthalate Metabolites and 5 Bisphenol Analogues in Human Urine.” Talanta 151: 224–233. 10.1016/j.talanta.2016.01.037. 26946031

[vms371209-bib-0018] Kang, J. W. , A. Y. Kim , H. Y. Chae , et al. 2023. “Forensic Analysis of Toxic Substances in Fatalities With Suspected Companion Animal Cruelty.” Korean Journal of Veterinary Research 63, no. 3: e21. 10.14405/kjvr.20230017.

[vms371209-bib-0019] Keating, M. P. , E. A. Saldo , J. L. Frair , S. A. Cunningham , R. Mateo , and D. S. Jachowski . 2024. “Global Review of Anticoagulant Rodenticide Exposure in Wild Mammalian Carnivores.” Animal Conservation 27, no. 5: 585–599. 10.1111/acv.12947.

[vms371209-bib-0020] Kohn, B. , C. Weingart , and U. Giger . 2003. “Haemorrhage in Seven Cats With Suspected Anticoagulant Rodenticide Intoxication.” Journal of Feline Medicine and Surgery 5, no. 5: 295–304. 10.1016/S1098-612X(03)00022-6. 12948505 PMC10822272

[vms371209-bib-0021] Kopanke, J. H. , K. E. Horak , E. Musselman , et al. 2018. “Effects of Low‐Level Brodifacoum Exposure on the Feline Immune Response.” Scientific Reports 8: 8168. 10.1038/s41598-018-26558-3. 29802369 PMC5970145

[vms371209-bib-0022] Lahmar, R. , P. Berny , T. Mahjoub , and S. Ben Youssef . 2019. “Animal Pesticide Poisoning in Tunisia.” Frontiers in Veterinary Science 6: 369. 10.3389/fvets.2019.00369. 31750320 PMC6848385

[vms371209-bib-0023] Lefebvre, S. , I. Fourel , S. Queffélec , et al. 2017. “Poisoning by Anticoagulant Rodenticides in Humans and Animals: Causes and Consequences.” In Poisoning—From Specific Toxic Agents to Novel Rapid and Simplified Techniques for Analysis, edited by N. Malangu . IntechOpen. 10.5772/intechopen.69955.

[vms371209-bib-0024] López‐Perea, J. J. , P. R. Camarero , I. S. Sánchez‐Barbudo , and R. Mateo . 2019. “Urbanization and Cattle Density Are Determinants in the Exposure to Anticoagulant Rodenticides of Non‐Target Wildlife.” Environmental Pollution 244: 801–808. 10.1016/j.envpol.2018.10.101. 30390453

[vms371209-bib-0025] Mahjoub, T. M. , E. Krafft , L. Garnier , et al. 2022. “Asymptomatic Anticoagulant Rodenticide Exposure in Dogs and Cats—A French and Belgian Rural and Urban Areas Study.” Frontiers in Toxicology 4: 907892. 10.3389/ftox.2022.907892. 35647575 PMC9131000

[vms371209-bib-0026] Mount, M. E. 1988. “Diagnosis and Therapy of Anticoagulant Rodenticide Intoxications.” Veterinary Clinics of North America: Small Animal Practice 18, no. 1: 115–130. 10.1016/S0195-5616(88)50012-8. 3282375

[vms371209-bib-0027] Mueller, C. A. , W. Weinmann , S. Dresen , A. Schreiber , and M. Gergov . 2005. “Development of a Multi‐Target Screening Analysis for 301 Drugs Using a QTRAP Liquid Chromatography/Tandem Mass Spectrometry System and Automated Library Searching.” Rapid Communications in Mass Spectrometry 19: 1332–1338. 10.1002/rcm.1934. 15852450

[vms371209-bib-0028] Nosal, D. G. , D. L. Feinstein , L. Chen , and R. B. van Breemen . 2020. “Separation and Quantification of Superwarfarin Rodenticide Diastereomers—Bromadiolone, Difenacoum, Flocoumafen, Brodifacoum, and Difethialone—in Human Plasma.” Journal of AOAC International 103, no. 3: 770–778. 10.1093/jaoacint/qsaa007. 33241367 PMC7372953

[vms371209-bib-0029] Nosal, D. G. , D. L. Feinstein , and R. B. van Breemen . 2021. “Chiral Liquid Chromatography–Tandem Mass Spectrometry Analysis of Superwarfarin Rodenticide Stereoisomers—Bromadiolone, Difenacoum, and Brodifacoum—in Human Plasma.” Journal of Chromatography B 1165: 122529. 10.1016/j.jchromb.2021.122529. PMC7875153 33486217

[vms371209-bib-0030] Peres, M. D. , S. Nascimento , and F. S. Pelição . 2019. “A New Clean‐Up Approach for Stomach Content Toxicological Analysis.” Forensic Science International 302: 109936. 10.1016/j.forsciint.2019.109936. 31493923

[vms371209-bib-0031] Sánchez‐Barbudo, I. S. , P. R. Camarero , and R. Mateo . 2012. “Primary and Secondary Poisoning by Anticoagulant Rodenticides of Non‐Target Animals in Spain.” Science of the Total Environment 420: 280–288. 10.1016/j.scitotenv.2012.01.028. 22326314

[vms371209-bib-0032] Seljetun, K. O. , E. Eliassen , R. Karinen , L. Moe , and V. Vindenes . 2018. “Quantitative Method for Analysis of Six Anticoagulant Rodenticides in Faeces, Applied in a Case With Repeated Samples From a Dog.” Acta Veterinaria Scandinavica 60: 3. 10.1186/s13028-018-0357-9. 29343296 PMC5772691

[vms371209-bib-0033] Tauer, D. 2025. “Anticoagulant Rodenticide Poisoning in Animals.” MSD Veterinary Manual. Accessed June 22, 2026. https://www.msdvetmanual.com/toxicology/rodenticide‐poisoning/anticoagulant‐rodenticide‐poisoning‐in‐animals.

[vms371209-bib-0034] Vandenbroucke, V. , A. Bousquet‐Mélou , P. De Backer , and S. Croubels . 2008. “Pharmacokinetics of Eight Anticoagulant Rodenticides in Mice After Single Oral Administration.” Journal of Veterinary Pharmacology and Therapeutics 31, no. 5: 437–445. 10.1111/j.1365-2885.2008.00979.x. 19000263

[vms371209-bib-0035] Vandenbroucke, V. , N. Desmet , P. De Backer , and S. Croubels . 2008. “Multi‐Residue Analysis of Eight Anticoagulant Rodenticides in Animal Plasma and Liver Using Liquid Chromatography Combined With Heated Electrospray Ionization Tandem Mass Spectrometry.” Journal of Chromatography B 869, no. 1–2: 101–110. 10.1016/j.jchromb.2008.05.011. 18502701

[vms371209-bib-0036] Waddell, L. S. , R. H. Poppenga , and K. J. Drobatz . 2013. “Anticoagulant Rodenticide Screening in Dogs: 123 Cases (1996–2003).” Journal of the American Veterinary Medical Association 242, no. 4: 516–521. 10.2460/javma.242.4.516. 23363284

[vms371209-bib-0037] Yang, W. , G. Hosgood , K. Luobikis , and A. Paul . 2018. “Agreement of Point‐of‐Care Prothrombin and Activated Partial Thromboplastin Time in Dogs With a Reference Laboratory.” Australian Veterinary Journal 96, no. 10: 379–384. 10.1111/avj.12746. 30255579

[vms371209-bib-0038] Zhu, L. , H. Yan , B. Shen , Y. Shi , M. Shen , and P. Xiang . 2013. “Determination of Bromadiolone and Brodifacoum in Human Hair by Liquid Chromatography/Tandem Mass Spectrometry and its Application to Poisoning Cases.” Rapid Communications in Mass Spectrometry 27, no. 4: 513–520. 10.1002/rcm.6477. 23322657

